# Exon 7 splicing of ERα predicts poor prognosis and increases phenotypic heterogeneity in luminal a subtype breast cancer

**DOI:** 10.1002/2211-5463.70215

**Published:** 2026-02-17

**Authors:** Long Wai Tsui, Taobo Hu, Thomson Christian Husein, Wai Hei Shek, Bingrong Chen, Depeng Wang, Shu Wang, Weichuan Yu, Xiaodan Fan, Mengping Long, Toyotaka Ishibashi

**Affiliations:** ^1^ Division of Life Science Hong Kong University of Science & Technology Hong Kong SAR; ^2^ Science for Life Laboratory, Department of Biochemistry and Biophysics Stockholm University Sweden; ^3^ GrandOmics Inc China; ^4^ Department of Breast Surgery Peking University People's Hospital China; ^5^ Department of Electronics and Computer Engineering The Hong Kong University of Science & Technology Hong Kong SAR; ^6^ Department of Statistics The Chinese University of Hong Kong Shatin Hong Kong SAR; ^7^ The Hong Kong University of Science and Technology Fok Ying Tung Research Institute Guangzhou China

**Keywords:** breast cancer, estrogen receptor, splicing, tumor development, tumor heterogeneity

## Abstract

ERαΔ7 is one of the most frequently observed splice variants of the *ESR1* gene in breast cancer but its functions remain largely unknown. Here we report that ERαΔ7 predicted poor survival in luminal A type breast cancer from TCGA data. ERαΔ7 occurred more frequently in luminal type breast cancer, with expression inversely correlating with *ESR1* expression. Functionally, ERαΔ7‐expressing MCF‐7 cells were found to have lower death rates at the expense of reduced growth and migration, while also being responsive to estrogen deprivation. Furthermore, molecular dynamics simulation showed that estradiol can bind to ERαΔ7. Our study indicated that ERαΔ7 might serve as a potential prognostic marker in Luminal A type breast cancer and has different functions from those of canonical ERα‐expressing cells.

## Graphical abstract

ERα splice variant ERα∆7 lacks the C‐terminus, and its expression may change phenotypes of breast cancers. Our results showed that ERα∆7 is found in the luminal A subtype, and elevated ERα∆7 levels are linked to improved cell survival with lower proliferation and migration. We propose that cells adjusting ERα∆7 levels via splicing may create a new level of phenotypic heterogeneity.

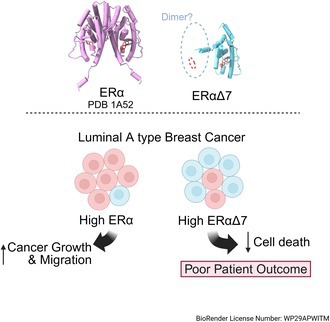

**Abbreviations**
CCK‐8: cell counting kit‐8
CRISPR: clustered regularly interspaced short palindromic repeats
DMEM: Dulbecco's modified Eagle medium
ER: estrogen receptor
ERE: estrogen responsive element
FACS: fluorescence‐activated cell sorting
FBS/PS: fetal bovine serum/Pen‐Strep
FPKM‐UQ: fragments per kilobase of transcript per Million mapped reads upper quartile
KO: knockout
MD: molecular dynamics
PCR: polymerase chain reaction
PSI: percent‐spliced in
RMSD: root‐mean‐square deviation
SDS/PAGE: sodium dodecyl‐sulfate polyacrylamide gel electrophoresis
SE: standard error
sgRNA: single‐guide RNA
TBS: tris‐buffered saline
TCGA: the cancer genome atlas
WT: wild‐type

Estrogen receptor (ER) is expressed in about 70% of all breast cancers and acts as an oncogenic driver as well as a therapeutic target [1, 2, 3]. The alpha subtype ERα (66 kDa) functions as a dimer with ERα or ERβ [4] and is important in both normal development and carcinogenesis, as it affects cell proliferation, survivability, plasticity, metabolism, and inflammation through two mechanistic pathways. The ERα in the nucleus acts as a transcription factor by binding to genomic estrogen receptor elements (EREs) to regulate thousands of genes including *CCND1*, *MYC*, *BCL2*, *PGR*, *GATA3*, *FOXA1*, and *ESR1* [5, 6, 7, 8, 9, 10, 11]. Alternatively, the ERα interacts with membrane or cytoplasmic signaling proteins to activate downstream pathways such as PI3K/Akt and MAPK/ERK [12, 13, 14, 15, 16, 17]. The summative effects of ERα on cancer are hence complex and heterogeneous; however, it is widely understood that estrogen receptor‐positive (ER^+^) cancers often recur and gain resistance toward endocrine therapies, even though they tend to be less aggressive than their ER^−^ counterparts [18, 19, 20, 21, 22, 23, 24].

The human *ESR1* gene transcript, consisting of eight exons, undergoes alternative splicing to produce splice variants of ERα [25, 26, 27, 28, 29, 30, 31, 32, 33]. This introduces an additional level of variety in the functions of ERα, as its variants often lose one or more functional domains. ERα consists of an AF‐1 domain, a DNA‐binding domain (DBD), a hinge region, and a ligand‐binding domain (LBD) with AF‐2 transactivation function. Since ERα plays roles in multiple cancer hallmarks, changes in splicing patterns can significantly alter cancer behavior. Understanding the functions and prognostic value of major splice variants enriches our understanding of ERα as a hallmark of oncogenesis.

ERα∆7 (51 kDa) is one of the most found splicing variants in breast cancer [25]. Skipping exon 7 leads to a frameshift and subsequent truncation, resulting in the production of a shorter 466 residue‐protein (Fig. 1A). The lost C‐terminal region contains the AF‐2 transactivation domain, a part of the ligand‐binding domain, and the ERα‐ERα dimerization interface. This is shown on our Alphafold2 structural predictions on residues 308–466 of ERα∆7 (Fig. 1B). In human patients, ERα∆7 expression was detected at roughly 1% of canonical ERα expression in both baseline and metastatic breast cancer patient tissue samples [25]. Functionally, an *in vitro* study found that ERαΔ7 acted in a dominant‐negative manner in regulating the ER pathway by inhibiting the binding of both ERα and ERβ to the EREs [35]. More recent studies showed a crucial role of ERαΔ7 in endometrial adenocarcinoma by determining the hormone sensitivity and efficiency of hormone treatment [36]. Additionally, ERαΔ7 was also associated with pathological invasiveness in lung adenocarcinoma [37].

**Fig. 1:**
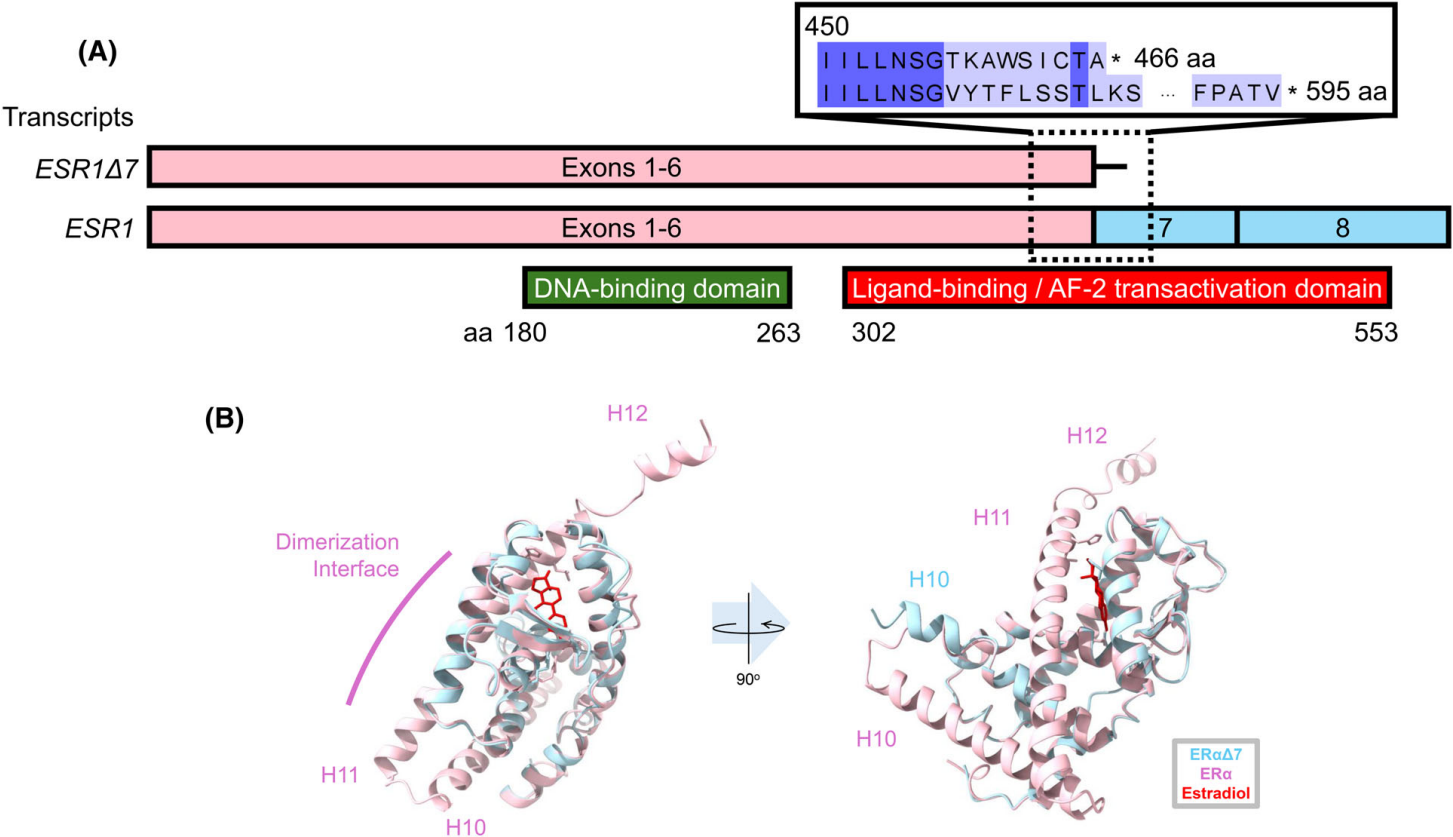
The sequence and predicted structure of ERαΔ7 aligned with canonical ERα. (A) Sequence alignment of ERαΔ7 with ERα. ERαΔ7 (51 kDa) is truncated at the C‐terminal at 466 amino acids. (B) The Alphafold2‐predicted structure of residues 308–466 of ERαΔ7 (blue) aligned with the structure of ERαΔ7 (PDB: 1A52, pink) [34].

Given the potential clinical significance of ERα∆7, we integrated the data analysis from TCGA with phenotypic assessments in luminal A subtype breast cancer MCF‐7 cells to investigate its prognostic value and functional impact. Here, our results showed that ERαΔ7 primarily existed in luminal A and B breast cancer subtypes. High ERαΔ7 levels predicted lower patient survival and lower *ESR1* transcript levels in the luminal A subtype. Furthermore, our ERαΔ7‐expressing MCF‐7 model exhibited a lower death rate with slower growth and migration, showing a phenotypic change compared to non‐ERαΔ7‐expressing cells. Finally, we provided evidence for estrogen sensitivity of ERαΔ7 through all‐atom molecular dynamics (MD) simulations and docking of estradiol binding to its ligand‐binding domain.

## Materials and methods

### TCGA analysis

The percent‐spliced‐in (PSI) index of ERα exon 7 in TCGA samples was downloaded from an open‐source database using the psichomics package in R and was used for the quantification of ERαΔ7 expression [38]. PSI value of ER exon 7 (hereon PSI (ERαΔ7)) in each sample was calculated by dividing the number of transcripts involving exon 7 with the total number of the transcript population of *ESR1*. A PSI value of 1 indicates constitutive inclusion of exon 7 in all transcripts, and PSI values smaller than 1 imply the skipping of exon 7 and the presence of ERαΔ7. The lower the PSI value is, the more abundantly ERαΔ7 was expressed relatively [39].

The Fragments Per Kilobase of transcript per Million mapped reads upper quartile (FPKM‐UQ) RNA‐seq data were downloaded and processed as described in our previous publications [40, 41, 42].

Cox proportional hazards regression was also performed to evaluate the association between exon 7 PSI and patient survival. In the univariate analysis, PSI values were treated as a continuous covariate in a Cox model for overall survival. For the multivariate Cox analysis, we included PSI (continuous) and adjusted for age at diagnosis, AJCC tumor stage, progesterone receptor (PR) status, and ESR1 expression. We assessed the proportional hazards (PH) assumption for each variable; since PR status violated the PH assumption, the Cox model was stratified by PR where necessary to obtain valid estimates. Hazard ratios (HR) with 95% confidence intervals were reported for the effect of PSI in both univariate and multivariate models.

The correlation analysis between the PSI value of ERαΔ7 and gene expression was evaluated with Pearson's correlation coefficient. Student's *t*‐test was utilized to compare PSI values between different groups. Patient survival was analyzed by the Kaplan–Meier estimate. *P*‐values were calculated as two‐sided.

### Tissue culture

Wild‐type MCF‐7 cells were purchased from ATCC (RRID: CVCL_0031). To generate cells expressing endogenous levels of ERαΔ7, a CRISPR‐Cas9 pipeline was used to insert the donor fragment ‘*ESR1* Exon6‐Exon8Δ‐StrepII::EF1a>GFP‐p2a‐*puroR’* in exon 6 of *ESR1* with homology directed repair of 500 bp homology sequence on both sides (Fig. 3A). The *ESR1* exon 6‐targeting sgRNAs used to generate breaks are 5′AAAATGTGTAGAGGGCATGG3′ and 5′CTCCCTGCAGATTCATCATG3′. sgRNAs were cloned into a pCasGuide plasmid (Origene, Vento, Netherlands; #SKU GE100002) and cotransfected with donor‐carrying pUC into wild‐type MCF‐7 using Lipofectamine 3000 (Invitrogen, Waltham, MA, USA; L3000008). Two heterozygous lines, *ESR1*∆7 (1) and (2), were selected by adding 1 μg·mL^−1^ puromycin (Thermo Fisher Scientific, Waltham, MA, USA; A1113803). Here, the Exon6‐Exon8Δ encodes for the C‐terminal of ERαΔ7 and the genomic exons 7 and 8 are not expressed due to a stop codon after the StrepII tag. The *ESR1* full length inserted line is prepared identically except the inserted fragment ‘*ESR1* Exon6‐Exon8Δ’ is replaced by Exon6‐Exon7‐Exon8 on the donor fragment. *ESR1*‐KO cells were generated using Estrogen Receptor 1 Human Gene Knockout Kit (Origene, #SKU KN213277) which destroys the promotor. All cells were verified by western blotting.

Wild‐type MCF‐7 cells were maintained in Dulbecco's modified Eagle medium (DMEM) (Thermo Fisher Scientific, #12100046) supplemented with 10% fetal bovine serum (FBS) (Thermo Fisher Scientific, #10270106) and 1% Pen‐Strep (PS) (Thermo Fisher Scientific, #15140122). CRISPR‐Cas9 edited cells were supplemented with an extra 0.5 μg·mL^−1^ puromycin. As phenol red acts as a weak estrogen and may affect assay results [43], a phenol red‐free DMEM (Thermo Fisher Scientific, #21063029)/10%FBS/1%PS (*yellow medium*) replaced the original medium four days before all assays. A 300 pg·mL^−1^ physiological concentration of β‐Estradiol (Sigma‐Aldrich, Burlington, MA, USA; E2257) is supplemented to the yellow medium unless otherwise specified. Cells were always grown at 37 °C/5% CO_2_ and mycoplasma‐free.

For western blotting, cells were trypsinized (Thermo Fisher Scientific, #25200072), lysed, resolved on 10% SDS/PAGE, and transferred to nitrocellulose. For anti‐StrepII blots, membranes were blocked in 1% BSA (Sigma‐Aldrich, #A9418) in TBS‐T (TBS + 0.1% Tween), incubated with anti‐StrepII (Sigma‐Aldrich, #71590–3, mouse mAb, 1 : 1000 in 1% BSA/TBS‐T), washed, reblocked in 5% BSA/TBS‐T, incubated with anti‐mouse‐HRP (Cytiva, Wilmington, DE, USA; #NA931V, 1 : 1000 in 1% BSA/TBS‐T), and washed. For anti‐ERα and anti‐H2B blots, membranes were blocked with 5% skim milk/TBS‐T, incubated with anti‐ERα (Abcam, Cambridge, UK; #ab79413, rabbit mAb, 1 : 500 in 3% BSA/TBS‐T) or anti‐H2B (ABClonal, Woburn, MA, USA; #A1958, rabbit pAb, 1 : 1000 in 3% BSA/TBST), washed, incubated with anti‐rabbit‐HRP (Cytiva, #NA934V lot 17136627, 1 : 3000 in 5% skim milk/TBS‐T), and washed. Anti‐StrepII and anti‐ERα blots were visualized using SuperSignal™ West atto (Thermo Fisher Scientific, #A38555), while the anti‐H2B blot was visualized using SuperSignal™ West pico plus (Thermo Fisher Scientific, #34580).

For genomic PCR, cells were trypsinized and sonicated in RIPA buffer. The genomic DNA is then harvested using routine DNA spin‐columns. Primers 5′GGGAGAGGAGTTTGTGTGCC3′ and 5′CAACTCAAGCACGAGGCG3′ were used to amplify regions between ESR1 exon 6 and EF1a with homemade Taq. Products were run on a routine 1% agarose for visualization. For Fig. S3, the primers used were 5′GGGAGAGGAGTTTGTGTGCC3′ and 5′GGCCATTTGATTCAATGCC3′ instead for a region between ESR1 exon 6 and intron 6.

### Phenotype assays

For colony formation assay, a total of 800 cells grown in yellow medium were seeded into each well of a 6‐well plate using a hemocytometer. The medium was partially replaced every 7 days for 19 days. Cells were then stained with 0.5% crystal violet and 6% glutaraldehyde, imaged, and analyzed in imagej/fiji (v. 2.3.0). Colony area was measured with the Analyze Particles plugin, and colony counts were obtained with the CellCounter plugin. Two repeats were performed with WT and *ESR1*‐full, and five repeats were performed with *ESR1*∆7 (1) and (2).

For survival assay, cells were cultured in yellow medium to achieve 50–80% confluency, then stained with 1 μg·mL^−1^ propidium iodide and Hoechst. Fluorescent images were acquired at 4× magnification on a Nikon Eclipse Ti microscope. For Fig. S8, the images were taken with and quantified by ImageXpress HCS.ai instead for higher throughput. imagej/fiji was used for analysis, with the Analyze Particles and CellCounter plugins to quantify propidium iodide‐positive and Hoechst‐positive cells. To account for inter‐batch differences, relative death was computed as death divided by the mean death of *ESR1*‐full on the same batch. For estrogen deprivation experiments, yellow medium with 30 or 0 pg·mL^−1^ estradiol was used instead. The experiment was repeated three times.

For cell counting assay, cells grown in yellow medium were seeded into 96‐well plates (using a hemocytometer) and designated as Day 0 and Day 4 groups. Upon reaching 50% confluency (Day 0), CCK‐8 (1 : 10 dilution) was added for 1 h at 37 °C, and absorbance was measured at 450 nm. The same procedure was repeated on Day 4. Relative absorbance was calculated as the Day 4 value divided by the average Day 0 value.

For EdU incorporation assay, the Click‐iT™ Plus EdU Alexa Fluor™ 647 Flow Cytometry Assay Kit (Thermo Fisher Scientific, #C10635) was used. Cells in yellow medium were grown to 20–40% confluency, treated with 10 μm EdU for 1 h, then trypsinized, fixed, and stained with Alexa 647 and 1 μg·mL^−1^ Hoechst following the manufacturer's instructions. A total of 10 000–30 000 cells per well were analyzed on a BD FACSAria™ III cell sorter, gated for single cells (SSC‐A/FSC‐A and DAPI‐W/DAPI‐A), and measured for EdU in the APC‐A channel. To account for differences between batches, relative EdU count was computed by dividing EdU count by the mean EdU count of *ESR1*‐full on the same batch. For tamoxifen‐supplemented sets, cells were treated at 1 μm tamoxifen 24 h ahead of EdU incorporation.

For wound‐healing assay, cells were cultured in yellow medium until 100% confluency. A 1.3 mm‐wide wound was created by scratching with a pipette tip. Wound closure was tracked at 0‐, 3‐, 6‐, 12‐, and 24‐h postinjury using a benchtop microscope.

Individual observations in all phenotype assays were subjected to a two‐sided Wilcoxon rank‐sum test (null hypothesis: identical distribution) using the stats package in R which eliminates day‐to‐day variations.

### Molecular dynamics and docking

All‐atom molecular dynamics (MD) simulations of estradiol‐bound ERα and ERαΔ7 were performed using GROMACS v2021.4 [44] with the CHARMM36m force field. The ERαΔ7 input model (residues 308‐466) was generated by AlphaFold2 [45], and estradiol was docked according to the established ERα structure (PDB: 1A52) [34] onto ERαΔ7, with the resulting RMSD between ERα and ERαΔ7 being 0.507 Å. The ERα input model was obtained by removing a monomer from 1A52. Both complexes were solvated in a 7.2 nm cubic TIP3 water box and neutralized with 0.15 M KCl using a Monte‐Carlo algorithm. The solvated system was then energy‐minimized with the deterministic steepest‐descent algorithm until the maximum force fell below 1000 kJ·mol^−1^·nm^−1^. Equilibration was performed under NVT conditions at 310.15 K using the V‐rescale thermostat for 125 ps, followed by NPT conditions at 1 bar using the Berendsen barostat for 125 ps. Production runs were carried out for 50 ns using a 2 fs timestep, with pressure maintained by the Parrinello‐Rahman barostat. Long‐range electrostatics were treated with Particle‐Mesh Ewald (1.2 nm cutoff), and all hydrogen‐involving bonds were constrained with the LINCS algorithm. Snapshots were recorded every 10 ps, and the first 10 ns was excluded from analysis, yielding 4000 frames. RMSD time‐course of the estradiol ligand was extracted using the ‘gmx rms’ command. Every alternate frame of four trajectories from each of ERα and ERα∆7 showing stable estradiol binding were selected for MM‐PBSA (gmx_MMPBSA v. 1.6.4 + 2.g1444795e) binding free energy (ΔG) determination. Here, ΔG = ΔEMM + ΔGsolv, where ΔEMM comprises van der Waals and electrostatic energies, and ΔGsolv comprises polar and nonpolar solvation contributions. Entropic contributions were not included.

Docking analysis was performed using a locally compiled build of AutoDock Vina (commit dbf9226‐mod) [46, 47]. The receptor and estradiol input structures were identical to those used in the GROMACS simulations, except that they were separated into individual files. The estradiol structure was energy‐minimized using Open Babel's built‐in conjugate gradient method with the MMFF94 force field, then docked into the receptor. The docking search space was defined as a cubic box with side lengths of 25 Å, centered at the center of mass of estradiol's original coordinates as determined from the crystal structure (PDB ID: 1A52) [34]. Upon successful docking with correct ligand orientation in the receptor binding pocket, binding energies were extracted. The final binding energy reported by AutoDock Vina accounts for receptor‐ligand interaction energy minus the ligand's internal torsional strain energy. Autodock Vina binding energies were subjected to the Wilcoxon rank‐sum test as described above.

## Results

### ERαΔ7 analysis in TCGA reveals prognostic value in luminal a breast cancer

**Fig. 2:**
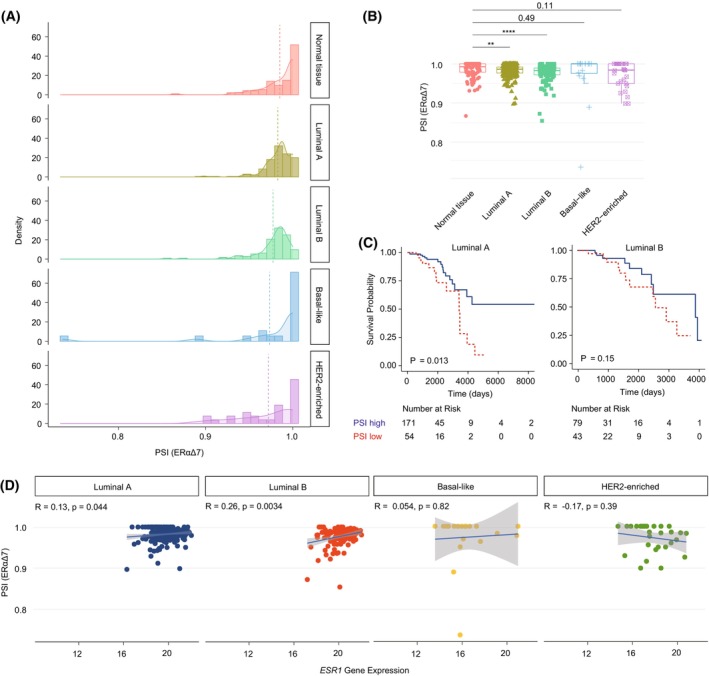
Bioinformatics analysis of ERαΔ7 using TCGA. (A) Distribution of PSI (ERαΔ7) in different breast cancer subtypes, and (B) the quantification. Error bar = 1.5*(Interquartile range). *P*‐values were calculated using two‐sided Wilcoxon rank‐sum tests for the indicated pairwise comparisons. (C) Comparison of survival between high PSI (ERαΔ7) and low PSI (ERαΔ7) groups. The cutoff of PSI = 0.976 was defined from the median PSI of the luminal A subtype. Note that a higher value of PSI means a lower abundance of ERαΔ7 and vice versa. *P*‐values were calculated using the log‐rank (Mantel–Cox) test. (D) Correlation of PSI (ERαΔ7) with *ESR1* gene expression in different breast cancer subtypes. Correlation was assessed using two‐sided Pearson correlation (reported as R with corresponding *P*‐value). Asterisks: ns, *P* ≥ 0.05; *P < 0.05; **P < 0.01; ***P < 0.001; ****P < 0.0001.

We analyzed the Percent‐Spliced In (PSI) values of *ESR1* exon 7 to evaluate ERαΔ7 expression across 112 normal breast tissues and 510 breast cancer samples from the TCGA database. PSI values close to 1 indicate full exon 7 inclusion (low ERαΔ7), while lower values suggest exon skipping (higher ERαΔ7). Across breast cancer subtypes, density plots showed PSI values near 1 in normal tissues, basal‐like, and HER2‐enriched subtypes, indicating minimal ERαΔ7 expression. In contrast, over half of Luminal A and Luminal B tumors had PSI values below 1, with median values of 0.983 and 0.977, respectively, suggesting relatively elevated ERαΔ7 expression (Fig. 2A, B, Fig. S1).

We compared survival with the mean PSI = 0.976 of luminal A subtype as a cutoff, dichotomizing all luminal A and B subtype samples into ‘High’ (PSI > 0.976, *n* = 211) and ‘Low’ (PSI < 0.976, *n* = 60) groups. Kaplan–Meier log‐rank test showed that in luminal A patients, lower PSI (higher ERαΔ7) was significantly associated with poorer overall survival (*P* = 0.013), not seen in the luminal B type (Fig. 2C). Since dichotomizing ERαΔ7 PSI at several cutoffs yielded imprecise multivariable Cox estimates (*P* = 0.475 for mean, *P* = 0.861 for median, and *P* = 0.197 for maxstat), we further analyzed the luminal A subtype data using ERαΔ7 PSI as a continuous predictor. Univariate Cox provided a hazard ratio of 0.71 (*P* = 0.034, 95% CI 0.52–0.97). Multivariate Cox adjusted for age, AJCC stage I–IV (orthogonal polynomial), PR status, and *ESR1* expression gave a hazard ratio of 0.71 (*P* = 0.070, 95% CI 0.49–1.03) when nonstratified; and a hazard ratio of 0.70 (*P* = 0.068, 95% CI 0.48–1.03) when PR is stratified for fulfilling the proportional hazards (PH) assumption. Further analysis of other clinicopathological features, including age at diagnosis, ER and PR status, histological subtype, and clinical stage, revealed no significant differences between the high and low PSI groups (Table S1). These collectively show that higher ERαΔ7 associates with worse survival in luminal A subtype breast cancer.

Furthermore, stratification by estrogen receptor (ER) immunohistochemistry (IHC) revealed that tumors with high ER expression (90–99%) had significantly lower PSI values than ER‐negative tumors, indicating higher ERαΔ7 expression in ER‐rich cancers, while no significant differences were seen across progesterone receptor (PR) IHC levels (Fig. S2). An inverse correlation between PSI values and *ESR1* mRNA levels was observed in luminal A and B subtypes (Fig. 2D), implying that increased ERαΔ7 expression may suppress *ESR1* transcription, potentially acting as a dominant‐negative variant. These together suggest that ERαΔ7 expression, as measured by exon 7 PSI values, could serve as an important prognostic marker in luminal A breast cancer, but we note that a bigger cohort is necessary to confirm whether ERαΔ7 serves as an independent prognostic marker.

### ERα∆7^+^ MCF‐7 cells demonstrate different hallmark phenotypes

**Fig. 3:**
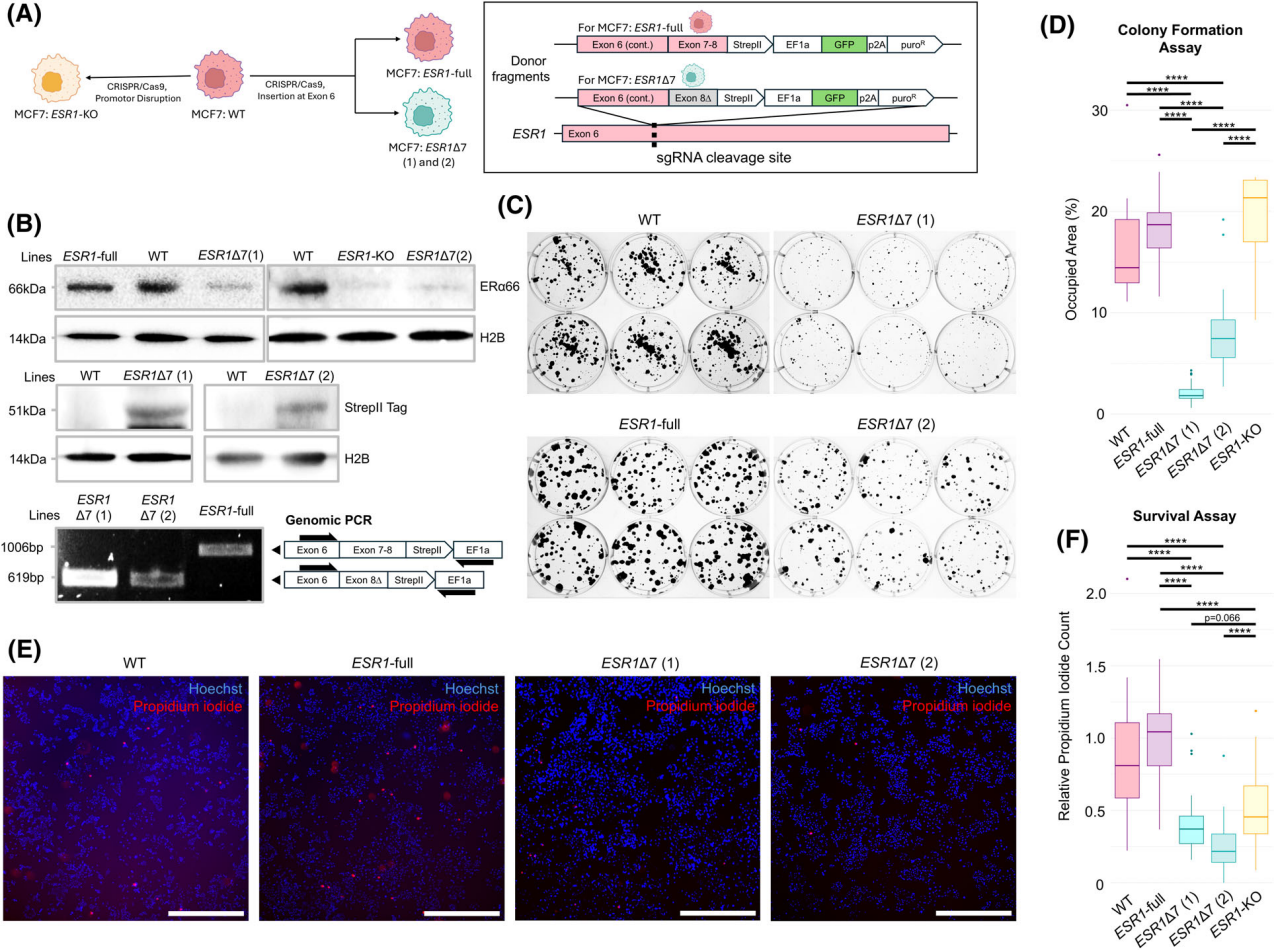
Phenotypes of ERαΔ7‐expressing MCF‐7 cells. (A) Schematic showing (Left) CRISPR/Cas9‐based engineering of MCF‐7 to generate *ESR1*Δ7, *ESR1*‐full, and *ESR1*‐KO lines; (Right) the cassette to be inserted into exon 6 of the *ESR1* gene to generate *ESR1*Δ7 and *ESR1*‐full lines. BioRender license number: JZ29APV8XJ. (B) (Top, Mid) western blots of the lines using anti‐ERα (Top) and anti‐StrepII (Mid), respectively. (Bottom) Genomic PCR of the lines with the amplicon between exon 6 and the downstream inserted EF1a promoter. (C) Representative images of the colony formation assay, and (D) the quantification. *n* = 12 for WT and *ESR1*‐full, *n* = 27 and 30 for *ESR1*Δ7 (1) and (2), respectively, error bars = SE. Significances were calculated by the Wilcoxon rank‐sum test given by the rank‐sum statistic W. Detailed *P*‐values: WT vs *ESR1*Δ7 (1) *P* = 8.69e‐7, WT vs *ESR1*Δ7 (2) *P* = 8.32e‐6, *ESR1‐*full vs *ESR1*Δ7 (1) *P* = 8.69e‐7, *ESR1*‐full vs *ESR1*Δ7 (2) *P* = 3.30e‐6, KO vs *ESR1*Δ7 (1) *P* = 8.69e‐7, and KO vs *ESR1*Δ7 (2) *P* = 4.61e‐6. (E) Representative images of the survival assay. Dead cells were stained with propidium iodide, and all others counterstained with Hoechst. Scale bar denotes 1 mm. (F) The quantification of the survival assay. *n* = 36, 58, 24, and 35 for WT, *ESR1*‐full, *ESR1*Δ7 (1), and (2) respectively, error bars = SE. Significances were calculated by the Wilcoxon rank‐sum test given by the rank‐sum statistic W. Detailed p‐values: WT vs *ESR1*Δ7 (1) *P* = 7.16e‐7, WT vs *ESR1*Δ7 (2) *P* = 1.30e‐11, *ESR1‐*full vs *ESR1*Δ7 (1) *P* = 4.60e‐10, *ESR1*‐full vs *ESR1*Δ7 (2) *P* = 4.54e‐15, KO vs *ESR1*‐full *P* = 2.37e‐10, KO vs *ESR1*Δ7 (1) *P* = 0.066, and KO vs *ESR1*Δ7 (2) 6.17e‐7.

Given the differences in survival and expression patterns related to ERαΔ7, we wondered whether ERαΔ7 delivered a different function in cells. To investigate its cellular role, we established a CRISPR/Cas9‐engineered MCF‐7 model that endogenously expresses ERαΔ7 and is representative of the luminal A subtype. A truncated exon 8 with StrepII tag was inserted at the end of exon 6 of the native *ESR1* gene (Fig. 3A). Two clones, *ESR1*Δ7 (1) and (2), were selected. We also produced an *ESR1*‐expressing line, namely ‘*ESR1*‐full’, where a full‐length exon 7 and 8 with StrepII tag were inserted at the same position and express full‐length ERα, and an *ESR1*‐KO line to act as controls in phenotype assessments. The expression of ERαΔ7/ERα and the insertions were confirmed by western blots and genomic PCR, respectively (Fig. 3B, Fig. S3). MCF‐7 cells were used because they are luminal A subtype breast cancer, which models the observations in the TCGA analysis.

To test the growth effects of splicing variants, a colony formation assay was conducted on *ESR1*Δ7 (1), (2), *ESR1*‐full, *ESR1*‐KO, and wild‐type MCF‐7 lines. At physiological concentrations of estradiol, colony areas of *ESR1*Δ7 (1) and (2) were smaller than these of the wild‐type, *ESR1*‐full, and *ESR1*‐KO lines (Fig. 3C,D). We noticed that *ESR1*Δ7 (1) and (2) formed many small colonies that are not contributing to the area. This result may be an indication of high cell survival with delayed growth in *ESR1*Δ7 (1) and (2). We therefore looked into the survival of ERα∆7^+^ cells under more favorable conditions. At high confluency and physiological concentration of estradiol, we counted dead cells using propidium iodide (Fig. 3E,F). Results showed that the death rate of wild‐type and *ESR1*‐full cells typically was in the 0.5–1.5% range, while the death rates of *ESR1*Δ7 (1) and (2) were in the 0.1–0.5% range (Fig. S4), which is a threefold reduction in cell death and exceeds the survival improvement in *ESR1*‐KO cells. These results showed that enhanced survival is observed in ERα∆7^+^ cells.

**Fig. 4:**
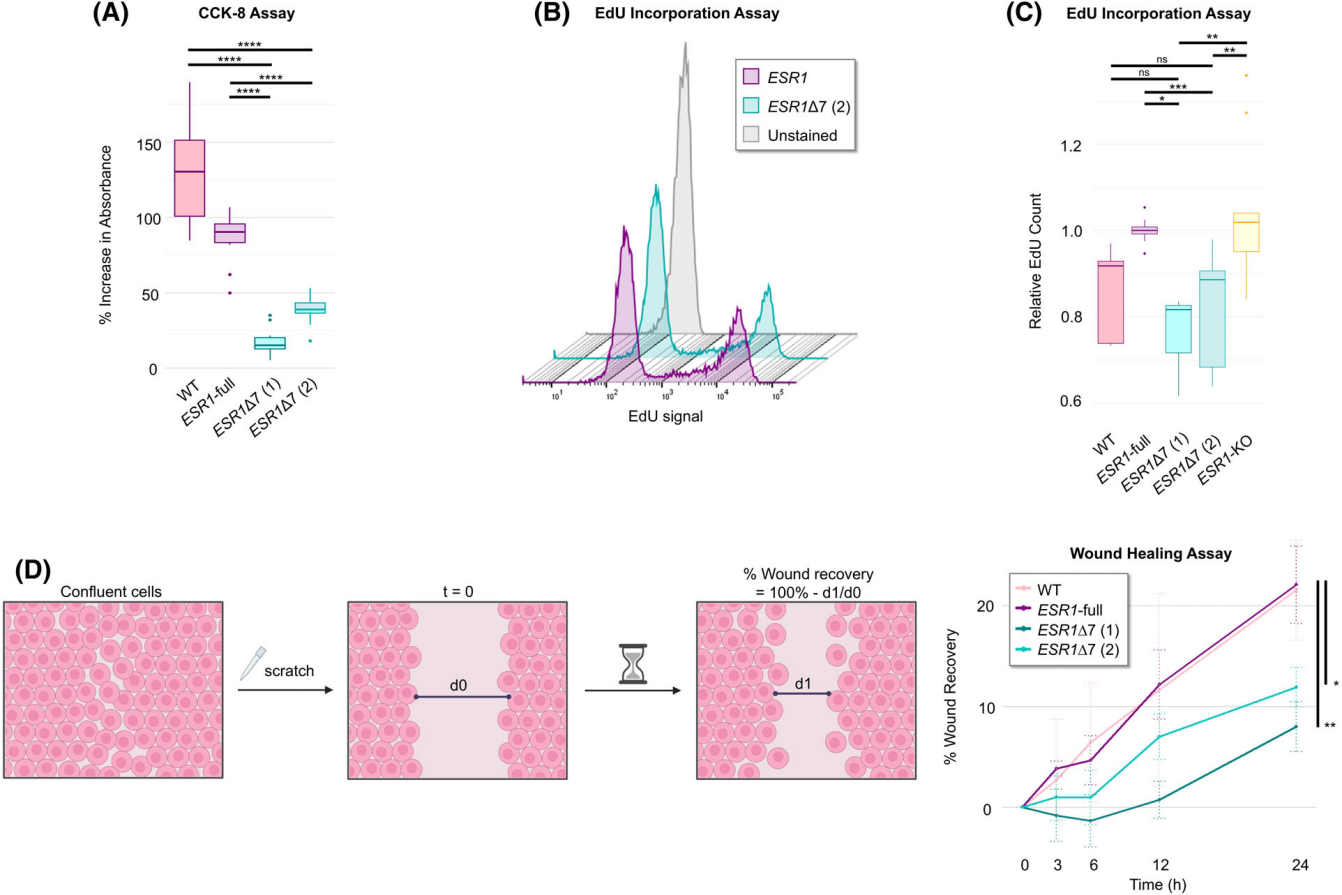
Diminished growth and migration of ERαΔ7‐expressing MCF‐7 cells. (A) Quantification of the increase in absorbance after 4 days of growth in the CCK‐8 assay. *n* = 12 for WT and ESR1, *n* = 18 for *ESR1*Δ7 (1) and (2), error bars = SE. Significances were calculated by the Wilcoxon rank‐sum test given by the rank‐sum statistic W. Detailed p‐values: WT vs *ESR1*Δ7 (1) *P* = 5.34e‐6, WT vs *ESR1*Δ7 (2) *P* = 5.33e‐6, *ESR1‐*full vs *ESR1*Δ7 (1) *P* = 5.34e‐6, and *ESR1*‐full vs *ESR1*Δ7 (2) *P* = 6.51e‐6. (B) After EdU incorporation, cells were sorted using FACS for EdU signal. Representative peaks from *ESR1*, *ESR1*Δ7 (2), and the unstained control are shown. (C) The quantification of the EdU incorporation assay. *n* = 5, 9, 3, and 8 for WT, *ESR1*‐full, *ESR1*Δ7 (1), and (2), respectively, error bars = SE. Significances were calculated by the Wilcoxon rank‐sum test given by the rank‐sum statistic W. Detailed p‐values: WT vs *ESR1*Δ7 (1) *P* = 0.37, WT vs *ESR1*Δ7 (2) *P* = 0.34, *ESR1‐*full vs *ESR1*Δ7 (1) *P* = 0.016, *ESR1*‐full vs *ESR1*Δ7 (2) *P* = 0.0012, KO vs *ESR1*Δ7 (1) *P* = 0.016, and KO vs *ESR1*Δ7 (2) *P* = 0.014. (D) Schematic showing the method and analysis of the wound‐healing assay and the quantification (*n* = 5). Error bars = Standard deviation. Significances were calculated by the Wilcoxon rank‐sum test given by the rank‐sum statistic W. BioRender license number: TP29AKA56U.

From the colony formation assay, ERα∆7^+^ cells may have a slower growth rate (Fig. 3C,D). To further investigate the effect of the growth rate, we tracked cell numbers at high confluency over four days using CCK‐8 (Fig. 4A, Fig. S5). They showed that while the wild‐type and *ESR1*‐full lines doubled in numbers over the period, both *ESR1*Δ7 (1) and (2) only increased by less than 1.5×. We also conducted a CCK‐8 assay at low confluency (starting from 2000 cells on day 0) and showed that the *ESR1*Δ7 (2) was growing slower despite not reaching significance at day 5 (Fig. S6). Moreover, to study the contribution for DNA replication, we proceeded to assess the S‐phase cell count using the EdU incorporation assay without synchronization (Fig. 4B, C). The data showed that the number of cells identified as positive signals of EdU is consistently 10–20% less than that of *ESR1*‐full and *ESR1*‐KO, despite not reaching significance with wild‐type. As cells incorporate EdU during DNA synthesis, such observations suggest that ERα∆7^+^ cells progress less into S‐phase; hence, they grow slower. We do not know whether it is the case that all cells grow slower or some cells opt for a slower cell cycle.

Finally, since ERα is involved in cell migration, we performed a wound‐healing assay to evaluate any changes in cell migration rates of ERα∆7+ cells (Fig. 4D, Fig. S9). ESR1Δ7 (1) and (2) cells began to migrate only 6 h after the wound was created. During the 12–24 h time frame, their migration rates were comparable to those of the wild‐type and ESR1‐full cell lines. This indicates that the expression of ERα∆7 may influence cell plasticity, but additional experiments focusing on cell morphology are necessary to confirm this.

### ERα∆7^+^ MCF‐7 cells responded to estrogen deprivation strategies

**Fig. 5:**
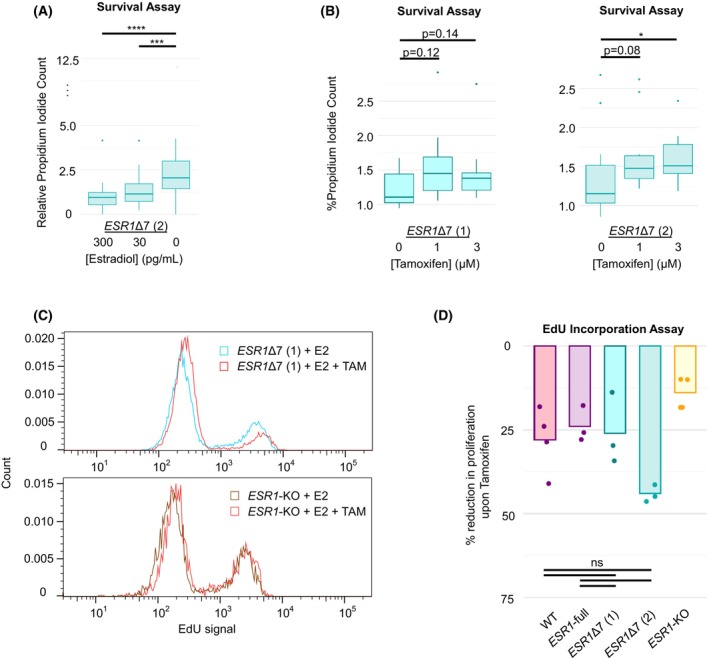
ERαΔ7‐expressing MCF‐7 responds to estrogen. (A, B) Quantification of the survival assay with (A) reduced estradiol concentration (*n* = 36, 35, 35 from left to right), and (B) supplemented tamoxifen (*n* = 8, 10, 9, 12, 10, 11 from left to right) in the medium. Error bars = SE. Significances were calculated by the Wilcoxon rank‐sum test given by the rank‐sum statistic W. Detailed *P*‐values: (A) [EST] 300 vs 0 pg·mL^−1^ *P* = 4.71e‐7, [EST] 30 vs 0 pg·mL^−1^ *P* = 3.2e‐4, (B) *ESR1*Δ7 (1) [TAM] 0 vs 1 μm *P* = 0.12, [TAM] 0 vs 3 μm *P* = 0.14, *ESR1*Δ7 (2) [TAM] 0 vs 1 μm *P* = 0.081, [TAM] 0 vs 3 μm *P* = 0.045. (C) After EdU incorporation, cells treated with tamoxifen were sorted using FACS for EdU signal. Representative peaks of ESR1Δ7 (1) and *ESR1*‐KO are shown. (D) The quantification of reduction of EdU‐positive cells when tamoxifen was added. *n* = 4 for WT and *ESR1*‐KO, *n* = 3 for *ESR1*‐full and *ESR1*Δ7 (1) and (2). Significances were calculated by the Wilcoxon rank‐sum test.

ER^+^ breast cancer often develops resistance toward endocrine therapies [48, 49]. ERα∆7 has a compromised ligand‐binding pocket and AF‐2 transactivation domain [4]. The lost helix 10 contains residues H524 and L525 which stabilizes the estradiol in the pocket. We investigated whether expression of ERα∆7 contributed to insensitivity toward estrogen deprivation. Surprisingly, in both survival and proliferation assays (Fig. 5, Figs S7, S8), we found that ERα∆7^+^ MCF‐7 cells responded to estrogen deprivation. In the survival assay (Fig. 5A), a complete removal of estradiol increased cell death by three folds, while addition of tamoxifen raised death by ~ 30% (Fig. 5B). In the proliferation assay (Fig. 5C,D), pretreatment with 1 μM tamoxifen for 1 h leads to a reduction in S‐phase entry to a similar extent with the wild‐type and *ESR1*‐full lines. These observations provide evidence that ERα∆7 alone is not sufficient to drive complete resistance.

**Fig. 6:**
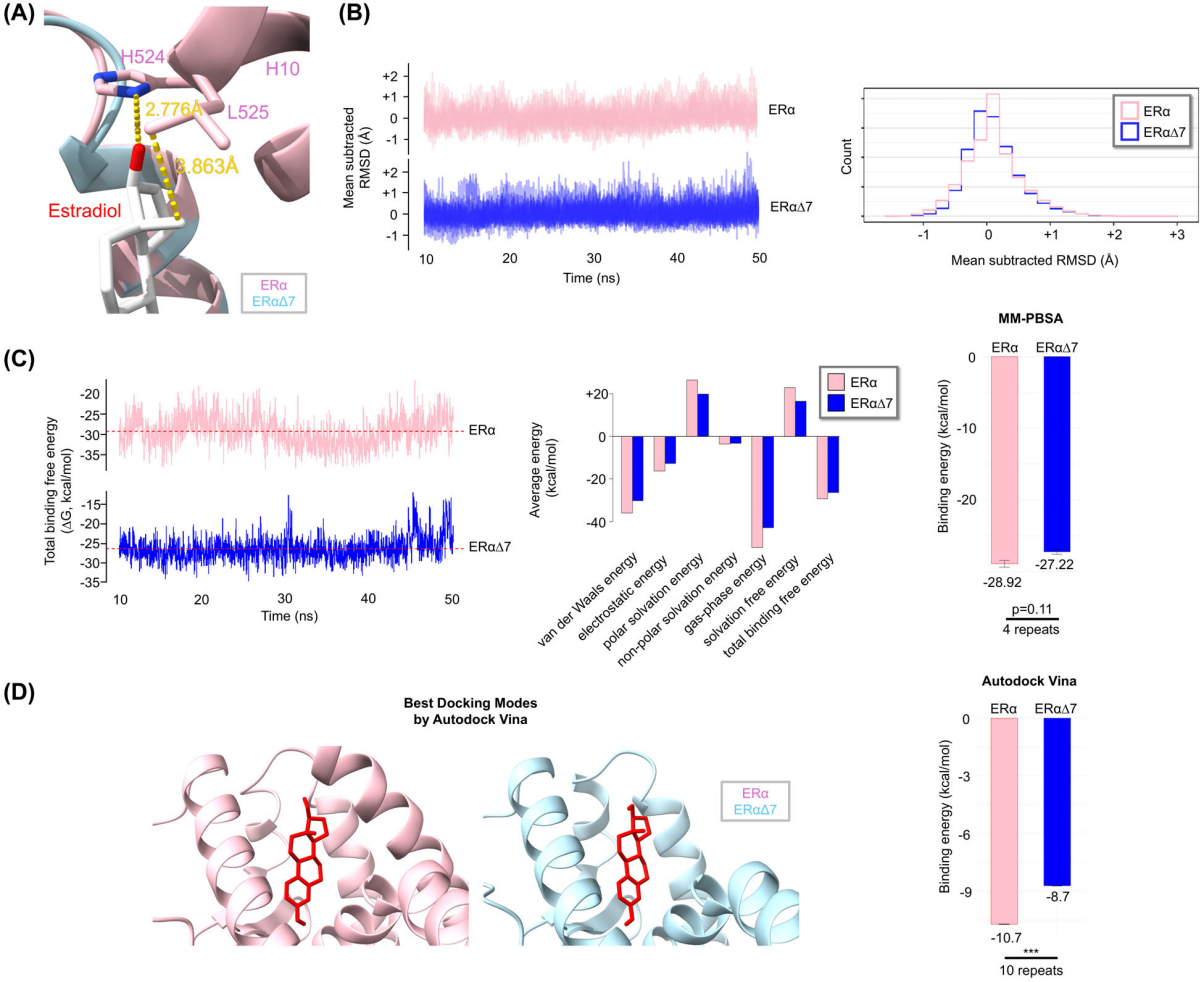
Molecular dynamics of estradiol‐bound ERαΔ7. (A) The helix 10 of ERα interacts with estradiol. This interaction is lost in ERαΔ7. ERαΔ7 structure is predicted by Alphafold2. (B) (Left) Mean subtracted RMSD of the estradiol bound to ERα (PDB 1A52) [34] and ERαΔ7 (Alphafold2‐predicted) over time (10 repeats overlaid). The first 10 ns are removed. (Right) The tallied mean subtracted RMSD of estradiol (10 repeats). (C) (Left) MM‐PBSA‐calculated binding energy of estradiol toward ERα (PDB 1A52) and ERαΔ7 (Alphafold2‐predicted) over time of a single trajectory, (Mid) its energy profile, and (Bottom) the quantification (*n* = 4, error bars = SE, significances calculated by the Wilcoxon rank‐sum test given by the rank‐sum statistic W). (D) (Left) Autodock Vina‐calculated best binding conformation of estradiol in ERα (PDB 1A52) and ERαΔ7 (Alphafold2‐predicted), and (Right) The binding energy comparison (*n* = 10, error bar = SE, significances calculated by the Wilcoxon rank‐sum test given by the rank‐sum statistic W. Detailed *P*‐values: *P* = 1.8e‐4).

If the estrogen‐binding pocket of ERα∆7 is compromised, we hypothesized that the ability of estradiol binding will be changed. We conducted all‐atom molecular dynamics simulations on estradiol inside either the ERα∆7 or ERα ligand‐binding domains using GROMACS. Since the structure for ERα∆7 is not available, we started with an Alphafold2‐predicted structure of ERα∆7 and docked estradiol according to the published structure of estradiol‐bound ERα ligand‐binding pocket [34] (Fig. 6A). Over a period of 50 ns and 10 repeats, estradiol stayed in both the ERα∆7 or ERα pockets for a similar extent of time, suggesting that estradiol‐ERα∆7 binding is not abolished (Fig. 6B, Video S1 (for ERα) and Video S2 (for ERα∆7)). We further calculated the binding energy of estradiol using MM‐PBSA. Results showed that the binding energy is lower in ERα∆7 than ERα by an average of 1.7 kcal·mol^−1^ which can be attributed to losing the two interactions on helix 10 (Fig. 6C). Strong binding between estradiol and ERα∆7 is still observed with a ∆G in the range of −20 to −30 kcal·mol^−1^. The high polar solvation energy for estradiol suggests that the energy penalty for estradiol to enter the aqueous environment contributes to its retainment in the pocket. We further tested whether a similar binding energy difference can be seen if the solvent is removed. Using Autodock Vina [46, 47], we docked estradiol in the absence of solvents and calculated the binding energy between estradiol and the receptor. Vina reported a 2.0 kcal·mol^−1^ reduction in binding energy, a proportion comparable to that in MM‐PBSA (Fig. 6D). These data suggest that ERα∆7 can still sequester estradiol and may contribute to the estradiol sensitivity of ERα∆7‐expressing cells.

## Discussion

ERαΔ7 PSI shows an association with overall survival in luminal A and provides incremental prognostic information beyond clinical covariates and *ESR1* expression (Fig. 2). Using an MCF‐7 based model, ERα∆7^+^ cells exhibited enhanced survival at the expense of reduced proliferation in the ER^+^ context. We also showed that ERαΔ7^+^ and *ESR1*‐KO MCF‐7 cells show distinct phenotypes and hence ERαΔ7 splicing is different from ER^−^ breast cancers. Since ERαΔ7 splicing is not a genomic process, the levels of ERα∆7 can likely be fine‐tuned, reversible, and spatio‐temporally distinct in the same tumor in adapting across microenvironments, allowing the tumor cell to choose between an ERα phenotype of high proliferation or an ERαΔ7 phenotype of high survival. We cocultured *ESR1*Δ7 cells with wild‐type cells and observed no competitive elimination of either kind, suggesting that cells with different ERα∆7 levels can coexist at the same time (data not shown). ERαΔ7‐associated survival may explain the poor survival and high recurrence in breast cancer, as cells expressing more ERαΔ7 can contribute to longer persistence in the body. A complete understanding on the heterogeneity of ERαΔ7 expression and its functional implications *in vivo* will require single‐cell spatio‐transcriptomic approaches.

We analyzed the relative expression of ERαΔ7 in breast cancer tissues from the TCGA database. Despite being one of the most frequently expressed splice variant of the *ESR1* gene, the relative expression of ERαΔ7 was low (as expected). However, it is possible that ERαΔ7 can affect the ER pathway activity as a minor component. The relative expression of ERαΔ7 inversely correlating with the expression of the *ESR1* gene could further contribute to the appearance of ERαΔ7 phenotype.

Our study raises critical questions about the upstream and downstream mechanisms governing ERαΔ7 expression and function. A key area of interest is how ERαΔ7 drives its distinct phenotypes, and we could speculate this from ERα36. ERα36 lacks the C‐terminal domain but is also N‐terminally truncated. It was previously reported that it functions via nongenomic signaling to interact with Akt/PI3K and MAPK/ERK pathways, with minimal transcriptional activities [50]. As Akt/PI3K and MAPK/ERK are both anti‐apoptotic, ERαΔ7 may follow a similar mechanism to promote cell survival while downregulating proliferative gene expression. This explanation is also in line with the reduction of *ESR1* transcript when ERαΔ7 is more abundant.

Our data suggest that ERαΔ7 expression alone does not confer resistance to endocrine therapy. A previous study [35] reported that ERαΔ7 suppresses estrogen‐mediated transcription in HeLa cells using a luciferase reporter system. As HeLa cells lack endogenous ERα, their system likely captured the isolated effects of ERαΔ7 in transcription. In contrast, our ERαΔ7‐expressing MCF‐7 cells retained estrogen responsiveness. We view this not as contradictory but as indicative of context‐specific functions. Consistent with our speculation that ERαΔ7 primarily acts through nongenomic pathways, MCF‐7 cells may provide additional epistatic factors that enable such signaling. Furthermore, our molecular dynamics simulations indicate that ERαΔ7 can still sequester estradiol. Further studies are needed to define any modulatory or cooperative roles ERαΔ7 may play in this nongenomic context.

Finally, splicing heterogeneity is inherently complex and spans multiple levels including variant and functional diversity, expression patterns, and cell types. In our system, we employed genomic editing to recapitulate nongenomic regulatory effects, leading to a higher proportion of ERαΔ7 (despite endogenous) than the estimated 1–2% (bulk) ERαΔ7/ERα ratio in patients. This also means the amounts of ERα levels may vary across our MCF‐7 lines leading to experimental artifacts. As the actual abundance of ERαΔ7 remains unknown in a single‐cell context, the clinical phenotypes may be more subtle than observed here.

## Conflict of interest

The authors have no conflict of interest.

## Author contributions

LWT and TI designed the cell culture and simulation experiments and wrote the paper. TH and ML designed and performed the bioinformatics analysis. LWT, TCH, WHS, and BC acquired and analyzed the data. DW, SW, WY, XF, and TI supervised and directed the project.

## Supporting information


**Table S1.** Patient clinicopathological features of the ERαΔ7 PSI high and low groups in Luminal A breast cancer patients.

**Fig. S1.** Quantification of PSI (ERαΔ7) distribution in ER‐expressing breast cancers only.
**Fig. S2.** Quantification of PSI (ERαΔ7) distribution against varying ER and PR expression levels determined via IHC.
**Fig. S3.** Genomic PCR on an amplicon between ESR1 exon 6 and intron 6.
**Fig. S4.** Quantification of replicates of individual survival assays.
**Fig. S5.** Distribution of absorbance at 450 nm in the CCK‐8 assay.
**Fig. S6.** Relative absorbance of the CCK‐8 assay when cells are plated at low densities.
**Fig. S7.** Quantification of individual survival assays with estrogen removed.
**Fig. S8.** Quantification of the survival assay of the ESR1 line with estrogen removed.
**Fig. S9.** Images of the wound‐healing assay with the wound sizes shown.

**Video S1.** All‐atom molecular dynamics simulation of ERα in complex to estradiol (PDB 1A52).

**Video S2.** All‐atom molecular dynamics simulation of ERα∆7 in complex to estradiol.

## Data Availability

Bioinformatics data were collected from the TCGA database. For these data, please contact Long M. at mengping.long@scilifelab.se.
