# Supplementary material for: Exon 7 splicing of ERα predicts poor prognosis and increases phenotypic heterogeneity in luminal a subtype breast cancer
Source: FEBS Open Bio. 2026 Feb 17;16(7):1400–13. doi: 10.1002/2211-5463.70215 (PMC13327064; doi:10.1002/2211-5463.70215)
Supplement: Supplementary file 1 — Table S1. Patient clinicopathological features of the ERαΔ7 PSI high and low groups in Luminal A breast cancer patients. [file FEB4-16-1400-s002.pdf]

List of Supporting Information

Supplementary Table S1

Supplementary Figures S1, S2, S3, S4, S5, S6, S7, S8, S9

Supplementary Videos S1, S2

Supplementary Table S1

Characteristics	PSI High	PSI Low	P
Age			0.341
<35	3 (1.8%)	2 (3.7%)	
35-49	34 (19.9%)	16 (29.6%)	
50-69	96 (56.1%)	27 (50.0%)	
70+	38 (22.2%)	9 (16.7%)	
AJCC Stage [a]			0.214
Stage I	46 (27.7%)	8 (16.3%)	
Stage II	82 (49.4%)	32 (65.3%)	
Stage III	36 (21.7%)	8 (16.3%)	
Stage IV	2 (1.2%)	1 (2.0%)	
Histology			0.774
Infiltrating Ductal Carcinoma	132 (77.2%)	45 (83.3%)	
Infiltrating Lobular Carcinoma	25 (14.6%)	5 (9.3%)	
Mixed Histology	7 (4.1%)	2 (3.7%)	
Others	7 (4.1%)	2 (3.7%)	
ER			0.420
10-19%	1 (1.5%)		
40-49%		1 (1.5%)	
50-59%	4 (5.9%)		
60-69%	3 (4.4%)	1 (4.3%)	
70-79%	8 (11.8%)	5 (21.7%)	
80-89%	8 (11.8%)	2 (8.7%)	
90-99%	44 (64.7%)	14 (60.9%)	
PR			0.325
<10%	9 (13.0%)	4 (16.7%)	
10-19%	5 (7.2%)	4 (16.7%)	
20-29%		1 (4.2%)	
30-39%	8 (11.6%)		
40-49%	6 (8.7%)	2 (8.3%)	
50-59%	4 (5.8%)	1 (4.2%)	
60-69%	4 (5.8%)	1 (4.2%)	
70-79%	11 (15.9%)	4 (16.7%)	
80-89%	3 (4.3%)	3 (12.5%)	
90-99%	19 (27.5%)	4 (16.7%)	
Methylation Cluster [b]			0.697
1	39 (22.9%)	12 (22.2%)	
2	65 (38.2%)	22 (40.7%)	
3	15 (8.8%)	2 (3.7%)	
4	48 (28.2%)	16 (29.6%)	
5	3 (1.8%)	2 (3.7%)	

Characteristics	PSI High	PSI Low	P
CN Cluster [b]			0.277
1	12 (7.4%)	7 (13.7%)	
2	82 (50.3%)	20 (39.2%)	
3	25 (15.3%)	7 (13.7%)	
4	33 (20.2%)	10 (19.6%)	
5	11 (6.7%)	7 (13.7%)	
miRNA Cluster [b]			0.050
1	15 (9.0%)	3 (5.6%)	
2	38 (22.9%)	7 (13.0%)	
3	5 (3.0%)	1 (1.9%)	
4	55 33.1(%)	13 24.1(%)	
5	4 (2.4%)	5 (9.3%)	
6	39 (23.5%)	18 (33.3%)	
7	10 (6.0%)	7 (13.0%)	

[a] AJCC: The American Joint Committee on Cancer;

[b] The clusters were previously defined by the Cancer Genome Atlas Network.

Table S1. Patient clinicopathological features of the ERαΔ7 PSI high and low groups in Luminal A breast cancer patients.

Supplementary Videos

Video S1

All-atom molecular dynamics simulation of ERα in complex to estradiol (PDB 1A52)[45].

Video S2

All-atom molecular dynamics simulation of ERαΔ7 in complex to estradiol. Structure from Alphafold2.
