# Supplementary material for: Exon 7 splicing of ERα predicts poor prognosis and increases phenotypic heterogeneity in luminal a subtype breast cancer
Source: FEBS Open Bio. 2026 Feb 17;16(7):1400–13. doi: 10.1002/2211-5463.70215 (PMC13327064; doi:10.1002/2211-5463.70215)
Supplement: Supplementary file 2 — Fig. S1. Quantification of PSI (ERαΔ7) distribution in ER‐expressing breast cancers only. Fig. S2. Quantification of PSI (ERαΔ7) distribution against varying ER and PR expression levels determined via IHC. Fig. S3. Genomic PCR on an amplicon between ESR1 exon 6 and intron 6. Fig. S4. Quantification of replicates of individual survival assays. Fig. S5. Distribution of absorbance at 450 nm in the CCK‐8 assay. Fig. S6. Relative absorbance of the CCK‐8 assay when cells are plated at low densities. Fig. S7. Quantification of individual survival assays with estrogen removed. Fig. S8. Quantification of the survival assay of the ESR1 line with estrogen removed. Fig. S9. Images of the wound‐healing assay with the wound sizes shown. [file FEB4-16-1400-s003.pdf]

Tsui LW, et al. Supplentary Figures S1, S2.

S1

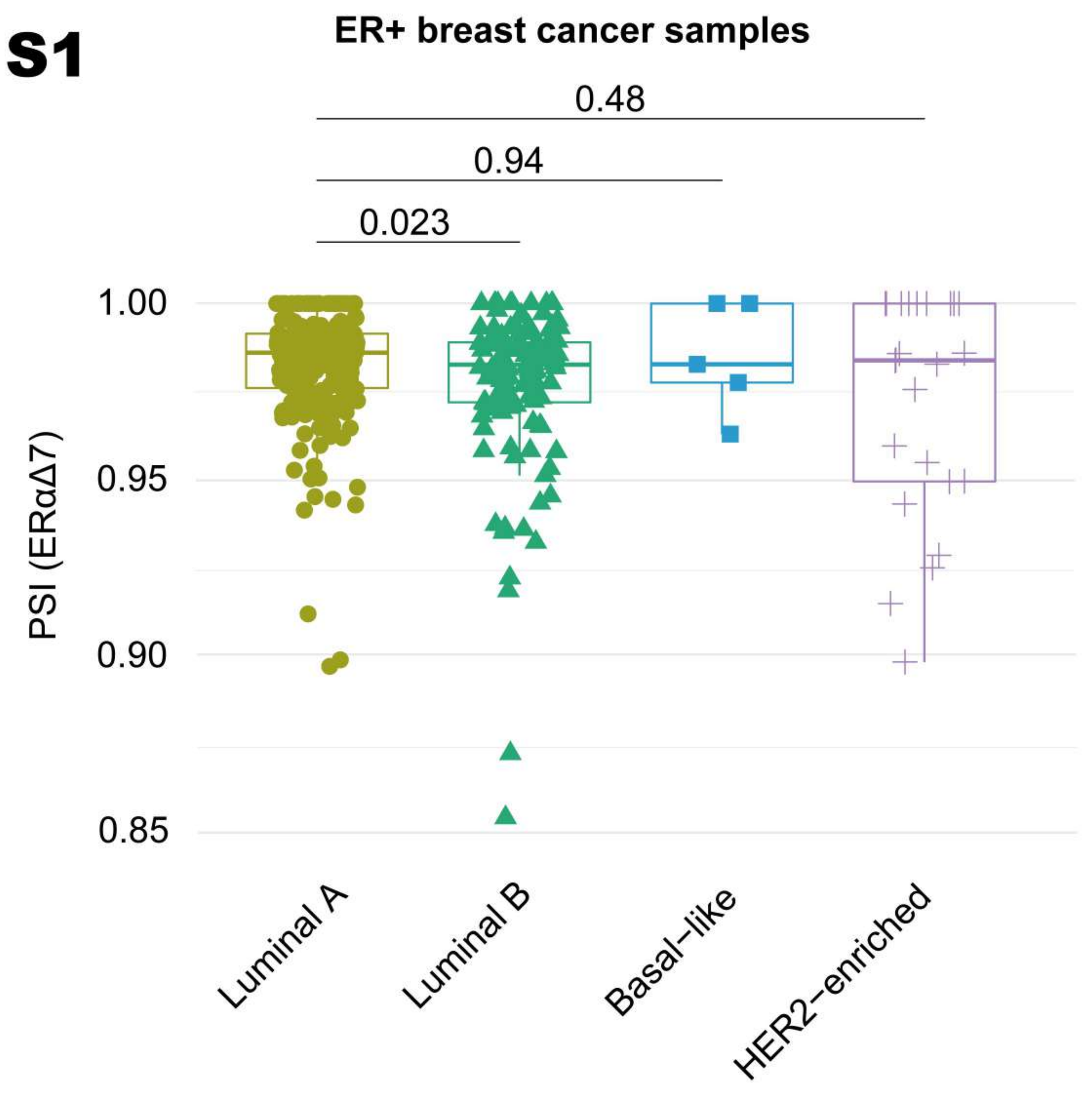

Figure S1
Quantification of PSI (ERαΔ7) distribution in ER-expressing breast cancers only. P-values were calculated using two-sided Wilcoxon rank-sum tests (Mann–Whitney U tests) for the indicated pairwise comparisons.

S2

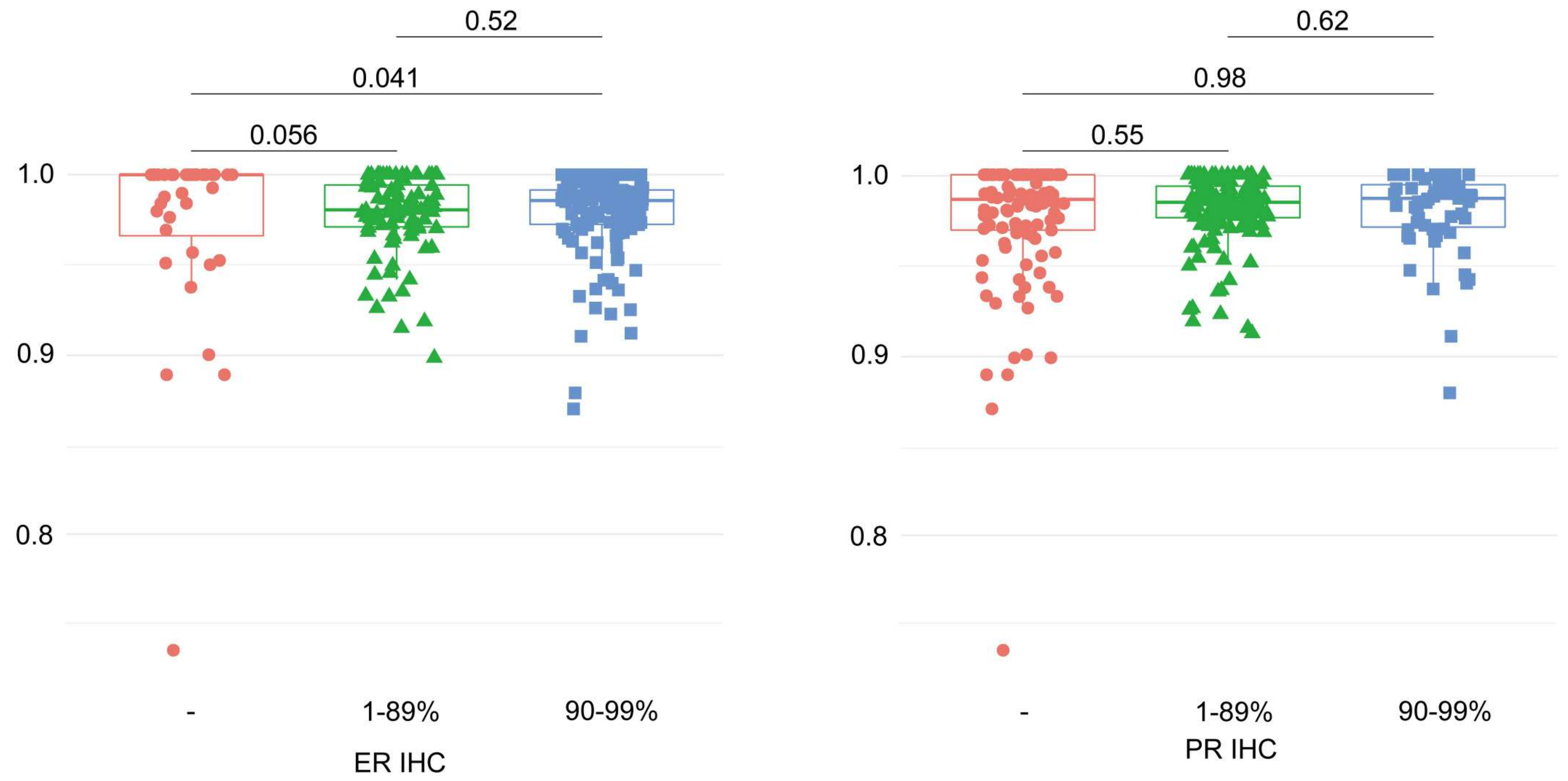

Figure S2
Quantification of PSI (ERαΔ7) distribution against varying ER and PR expression levels determined via IHC. P-values were calculated using two-sided Wilcoxon rank-sum tests (Mann–Whitney U tests) for the indicated pairwise comparisons.

Tsui LW, et al. Figure S3

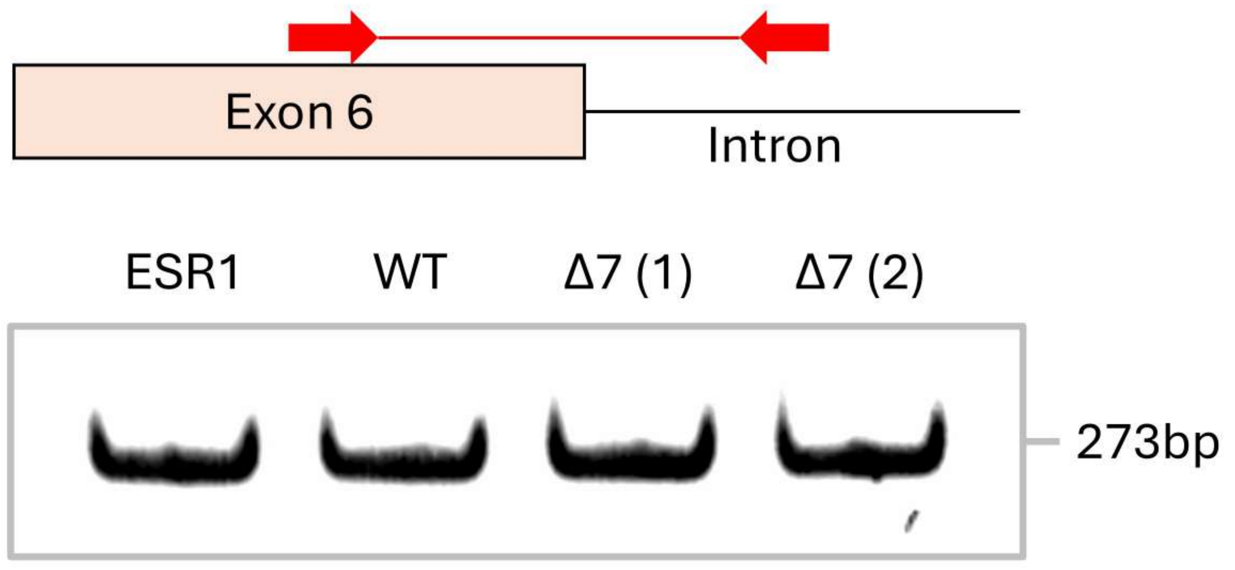

Figure S3
Genomic PCR on an amplicon between *ESR1* exon 6 and intron 6. This shows all *ESR1*Δ7 clones are heterozygous.

Tsui LW, et al. Figure S4

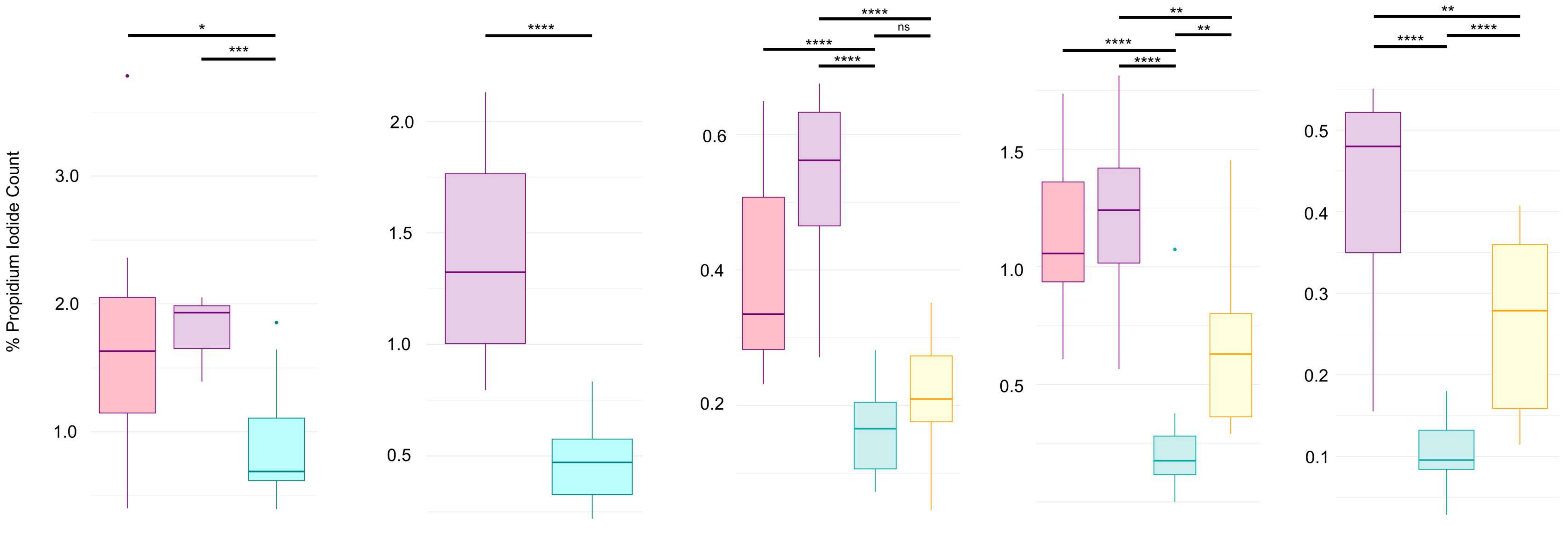

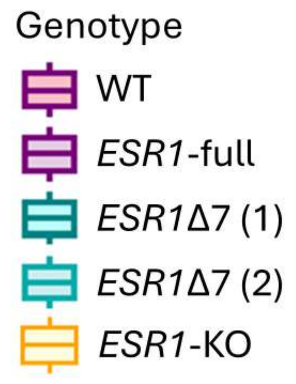

Figure S4
Individual survival assays. Genotypes in each figure are assayed in parallel. n=11-12.

Error bars = SE. Significances were calculated by the Wilcoxon rank-sum test given by the rank-sum statistic W.
Asterisks: ns, p ≥ 0.05; *, p < 0.05; **, p < 0.01; ***, p < 0.001; ****, p < 0.0001.

Tsui LW, et al. Figure S5

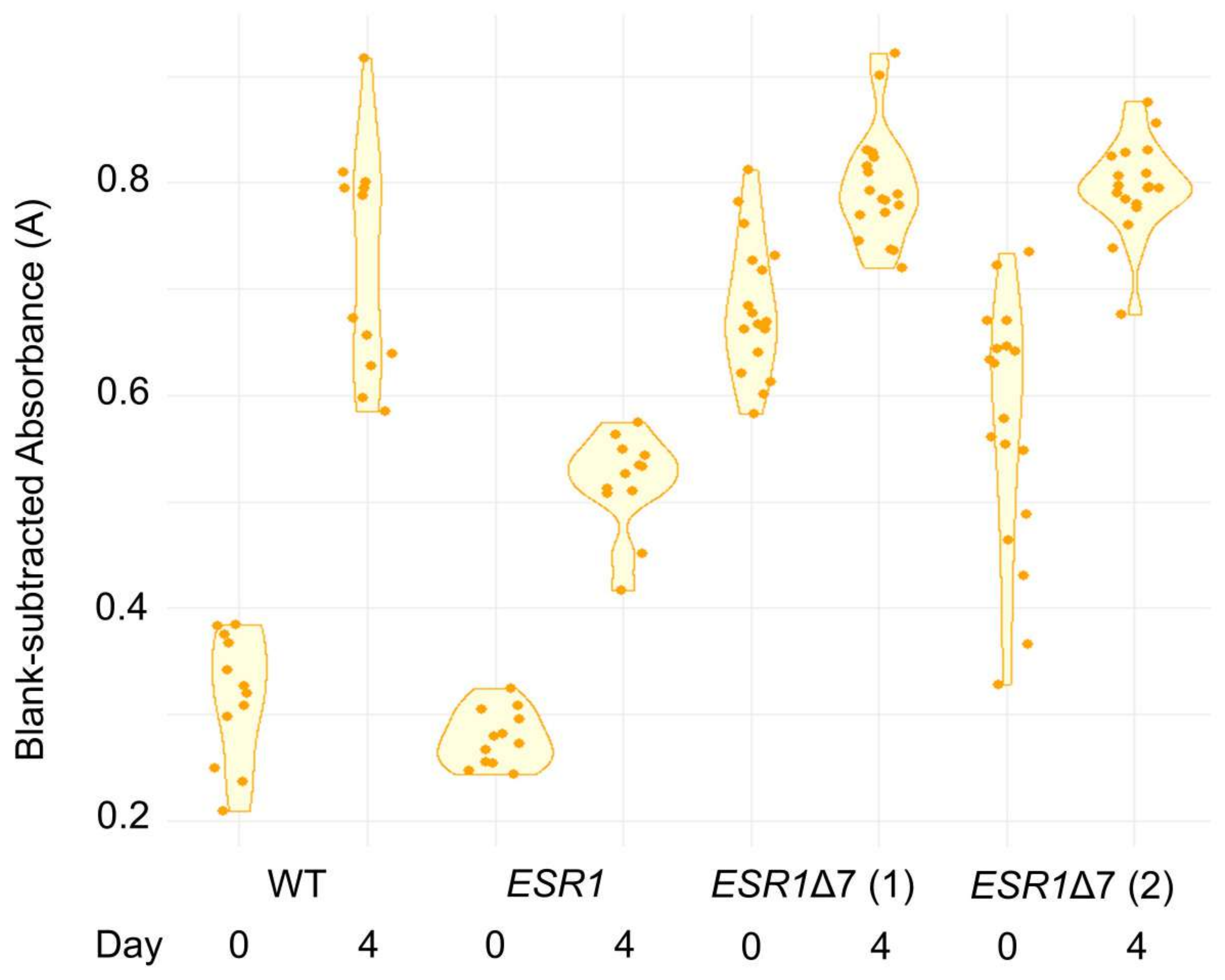

Figure S5
Distribution of absorbance at 450nm in the CCK-8 assay. n=12 for WT and ESR1, and 18 for ESR1Δ7 (1) and (2).

Error bars = SE. Significances were calculated by the Wilcoxon rank-sum test given by the rank-sum statistic W. Asterisks: ns, p ≥ 0.05; *, p < 0.05; **, p < 0.01; ***, p < 0.001; ****, p < 0.0001.

Tsui LW, et al. Figure S6

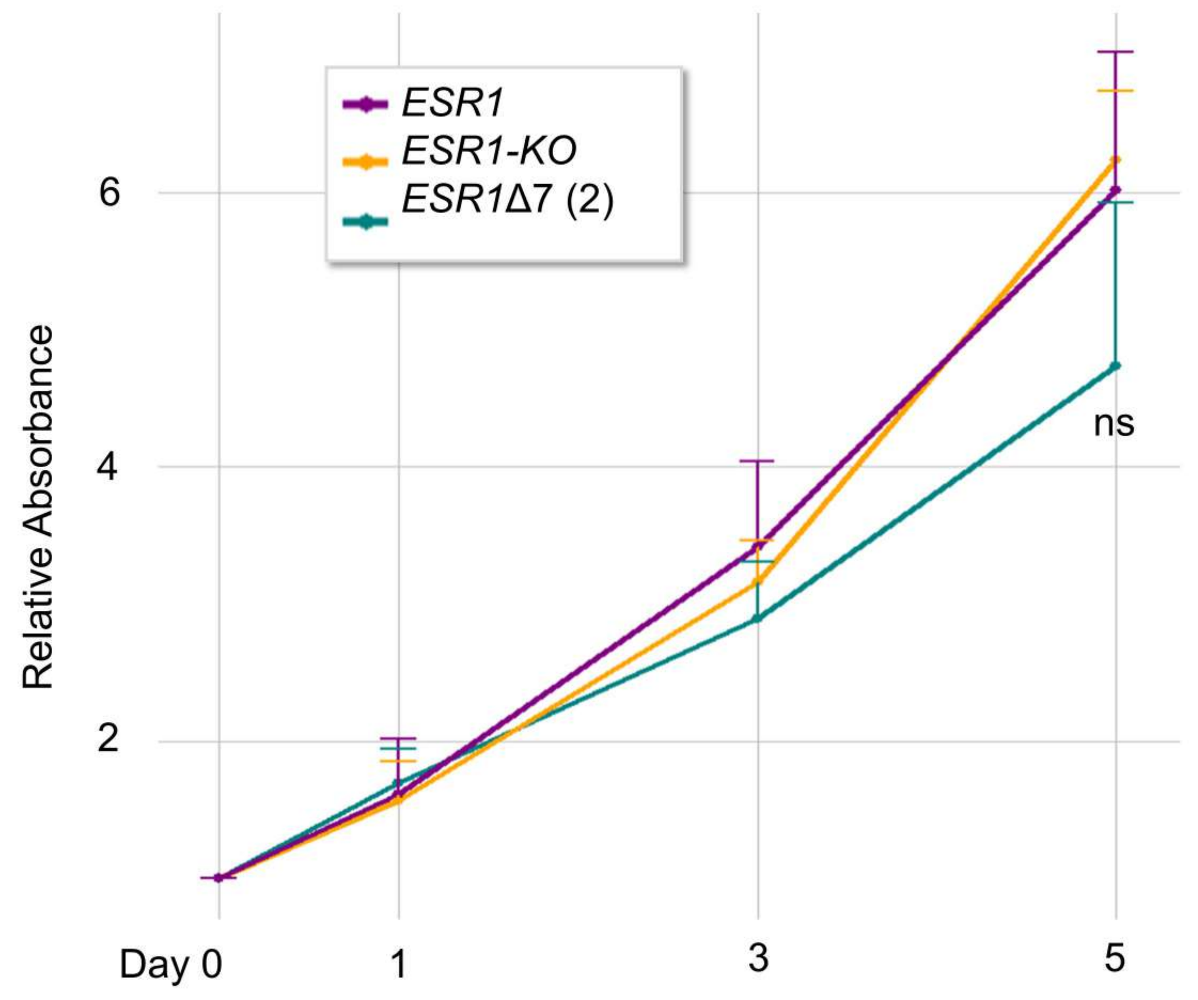

Figure S6
Relative absorbance of the CCK-8 assay when cells are plated at low densities. n=6.

Error bars = SE. Significances were calculated by the Wilcoxon rank-sum test given by the rank-sum statistic W.

Tsui LW, et al. Figure S7

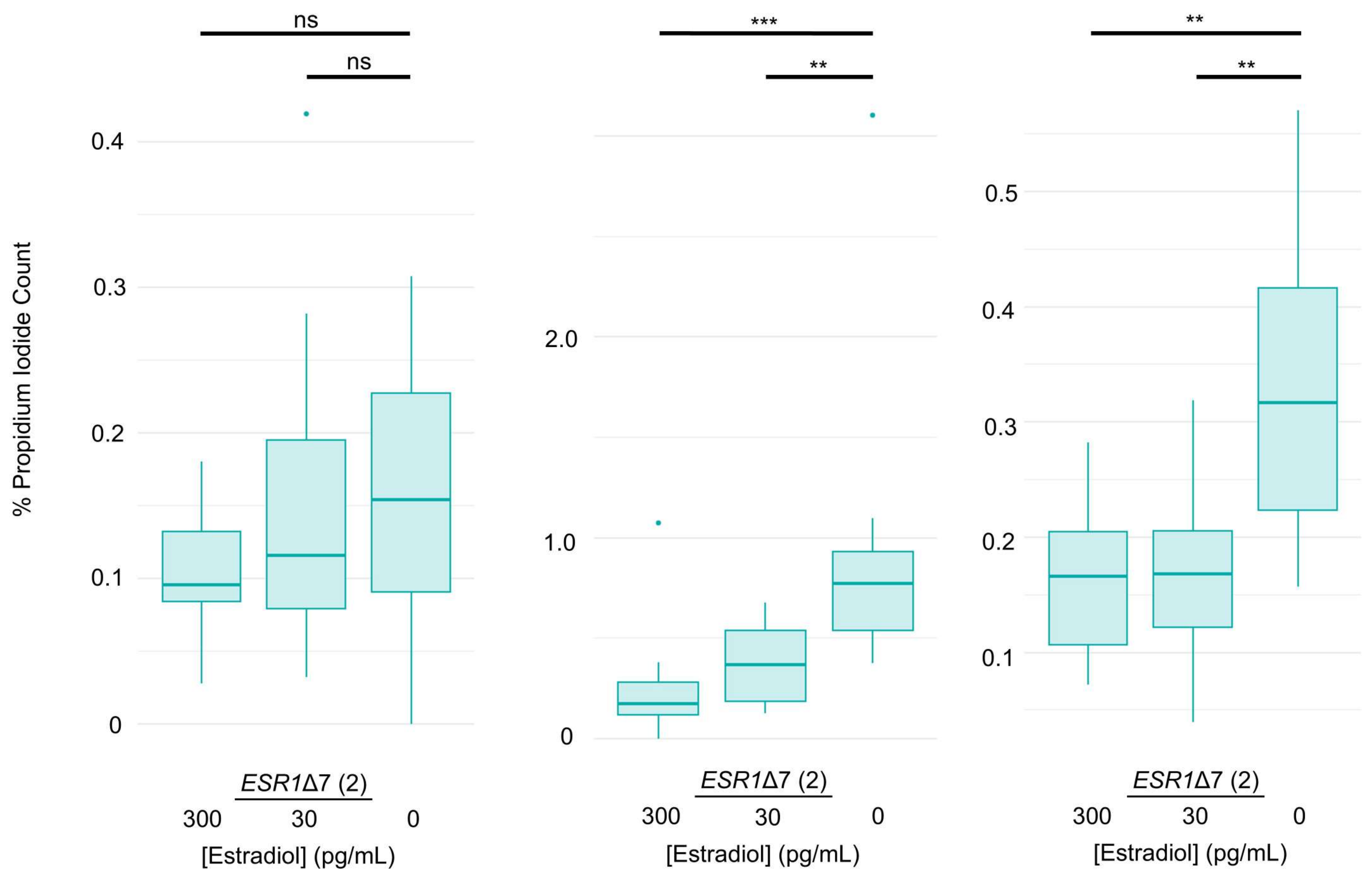

Figure S7
Individual survival assays with estrogen removed. Conditions in each figure are assayed in parallel. n=11-12.

Error bars = SE. Significances were calculated by the Wilcoxon rank-sum test given by the rank-sum statistic W. Asterisks: ns, $p \geq 0.05$; *, $p < 0.05$; **, $p < 0.01$; ***, $p < 0.001$; ****, $p < 0.0001$.

Tsui LW, et al. Figure S8

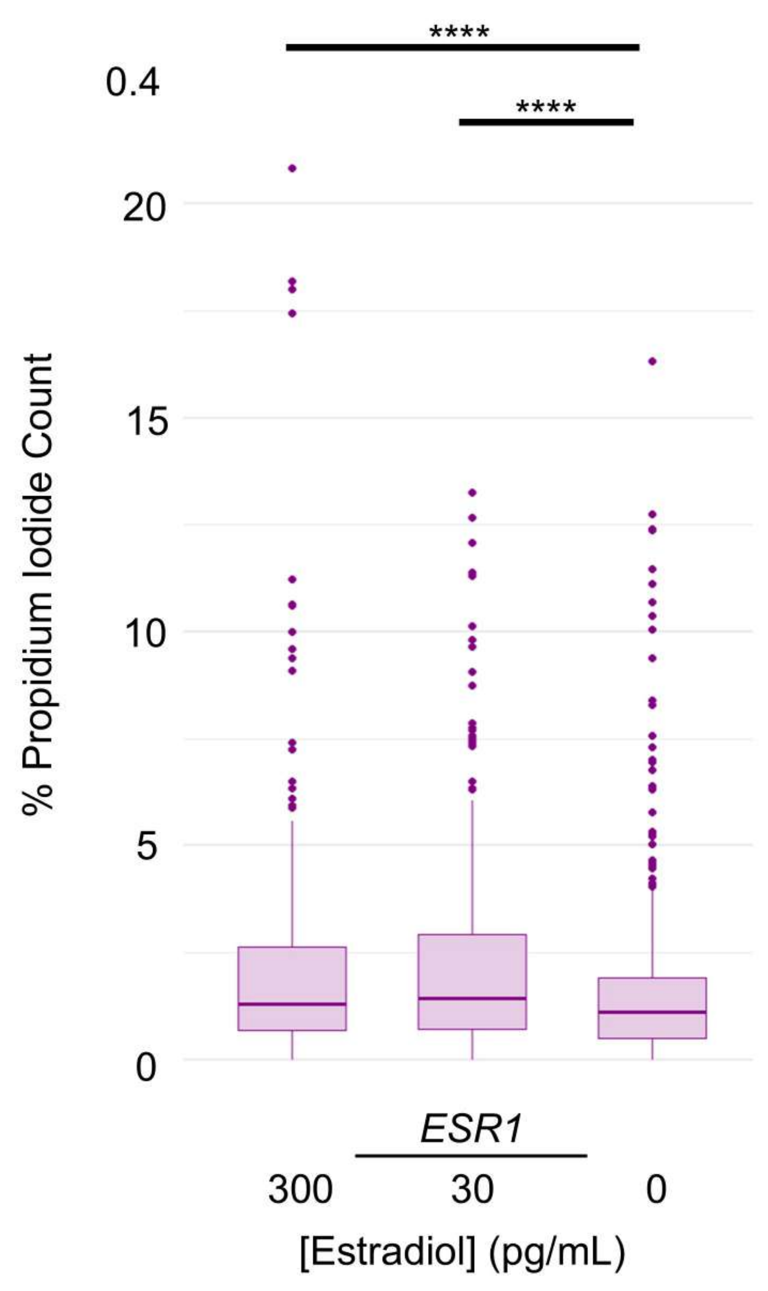

Figure S8
Survival assay of the *ESR1* line with estrogen removed. n=408.

Error bars = SE. Significances were calculated by the Wilcoxon rank-sum test given by the rank-sum statistic W. Asterisks: ns, $p \geq 0.05$; *, $p < 0.05$; **, $p < 0.01$; ***, $p < 0.001$; ****, $p < 0.0001$.

Tsui LW, et al. Figure S9

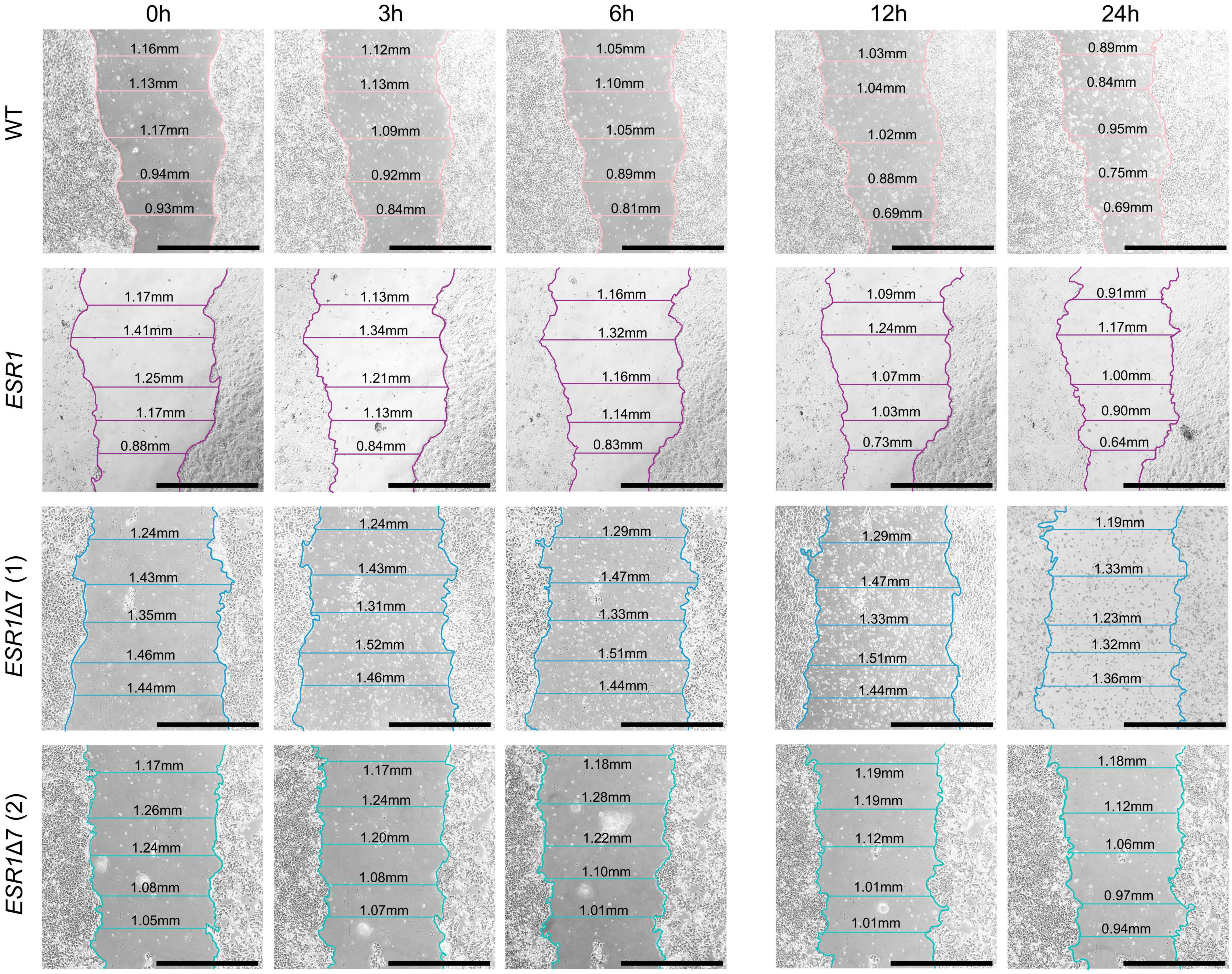

Figure S9
Images of the wound-healing assay with the wound sizes shown. Scale bar: 1mm.
